# Association of lifestyle with deep learning predicted electrocardiographic age

**DOI:** 10.3389/fcvm.2023.1160091

**Published:** 2023-04-24

**Authors:** Cuili Zhang, Xiao Miao, Biqi Wang, Robert J. Thomas, Antônio H. Ribeiro, Luisa C. C. Brant, Antonio L. P. Ribeiro, Honghuang Lin

**Affiliations:** ^1^ Department of Cardiology, The First Affiliated Hospital of Harbin Medical University, Harbin, China; ^2^ Innovation Research Institute of Traditional Chinese Medicine, Shanghai University of Traditional Chinese Medicine, Shanghai, China; ^3^ Department of Medicine, University of Massachusetts Chan Medical School, Worcester, MA, United States; ^4^ Department of Medicine, Division of Pulmonary, Critical Care & Sleep Medicine, Beth Israel Deaconess Medical Center, Boston, MA, United States; ^5^ Department of Information Technology, Uppsala University, Uppsala, Sweden; ^6^ Faculty of Medicine and Telehealth Center, Hospital das Clínicas, Universidade Federal de Minas Gerais, Belo Horizonte, Brazil

**Keywords:** biological age, deep learning, lifestyle, epidemiology—analytic (risk factors), electrocardiogram

## Abstract

**Background:**

People age at different rates. Biological age is a risk factor for many chronic diseases independent of chronological age. A good lifestyle is known to improve overall health, but its association with biological age is unclear.

**Methods:**

This study included participants from the UK Biobank who had undergone 12-lead resting electrocardiography (ECG). Biological age was estimated by a deep learning model (defined as ECG-age), and the difference between ECG-age and chronological age was defined as Δage. Participants were further categorized into an ideal (score 4), intermediate (scores 2 and 3) or unfavorable lifestyle (score 0 or 1). Four lifestyle factors were investigated, including diet, alcohol consumption, physical activity, and smoking. Linear regression models were used to examine the association between lifestyle factors and Δage, and the models were adjusted for sex and chronological age.

**Results:**

This study included 44,094 individuals (mean age 64 ± 8, 51.4% females). A significant correlation was observed between predicted biological age and chronological age (correlation coefficient = 0.54, *P* < 0.001) and the mean Δage (absolute error of biological age and chronological age) was 9.8 ± 7.4 years. Δage was significantly associated with all of the four lifestyle factors, with the effect size ranging from 0.41 ± 0.11 for the healthy diet to 2.37 ± 0.30 for non-smoking. Compared with an ideal lifestyle, an unfavorable lifestyle was associated with an average of 2.50 ± 0.29 years of older predicted ECG-age.

**Conclusion:**

In this large contemporary population, a strong association was observed between all four studied healthy lifestyle factors and deaccelerated aging. Our study underscores the importance of a healthy lifestyle to reduce the burden of aging-related diseases.

## Introduction

The worldwide population is becoming older. It is estimated that 1.5 billion people will be 65 years or over in 2050 (1). Aging is inevitable but people could age at different rates (2). Chronic inflammation and some underlying health conditions could contribute to accelerated aging (3). Chronological age is the age people usually refer to, which represents the amount of time from birth to a given time. In contrast, biological age represents the measurement of age based on different biomarkers and it is known to associate with different chronic diseases and all-cause mortality in later life independent of other risk factors (4, 5). Many different measures of biological aging have been proposed, such as epigenetic biomarkers (6, 7), clinical factors, sleep-based brain age (8), inflammatory biomarkers (9), and cardiovascular magnetic resonance imaging biomarkers (10).

The electrocardiogram (ECG) has been used in clinics to diagnose many different cardiovascular diseases for more than a century. For example, a long QT interval in ECG is known to associate with multiple heart diseases. Recently, deep learning models are being developed to diagnose diseases directly from ECG waveforms (11). Multiple studies have demonstrated the potential of ECG to diagnose atrial fibrillation and other types of heart diseases (12–15). Methods are also being developed to predict biological age from these ECG waveforms (referred as ECG-age) (16–18). The discrepancies between chronological age and ECG-age might serve as a biomarker for underlying diseases and all-cause mortality (17, 18).

A good lifestyle is known to benefit health. It includes avoiding smoking, being physically active, no or moderate alcohol consumption, and having a healthy diet. The combination of multiple good lifestyle behaviors and genetic factors are also associated with reduced risk of cardiovascular disease (19), diabetes (20), and all-cause mortality (21).

This study aims to predict ECG-age and assess its association with lifestyle factors in the UK Biobank, a large prospective population-based cohort based in UK. We also examined the association of ECG-age with prevalent cardiovascular diseases and risk factors.

## Methods

### Study samples

The UK Biobank is a nationwide, population-based prospective study (22). More than 500,000 participants aged 40–69 years were recruited during 2006–2010 at 22 assessment centers throughout the UK (23). The objective of the UK Biobank is to understand both genetic and environmental determinants of common life-threatening and disabling diseases (24). A variety of data have been collected from participants, including questionnaires, physical measurements, as well as different biological samples like blood, urine, and saliva (25). The study is embedded within the UK's National Health Service, so that routine medical records are extractable for different disease outcomes. The UK Biobank study has been approved by the North West Multi-center Research Ethics Committee (MREC). All participants provided written informed consent to participate. The current analysis was approved by the Research Ethics Committee (REC) under the application number 76,269.

### ECG collection

A standardized resting 12-lead ECG was conducted at a UK Biobank Imaging Assessment Centre. The ECG was obtained before and during a submaximal exercise test on a stationary bicycle. Participants were asked to lie on the same couch used to perform the carotid ultrasound measurement. ECG GE Cardiosoft program is loaded into the workstation and used to record ECG. We retrieved the ECG data in extended markup language (XML) files (Data-Field 20205). More information could be found at https://biobank.ctsu.ox.ac.uk/crystal/field.cgi?id=12323.

The ECG leads were recorded with a 500 Hz sampling frequency for 10 s. The recordings were then re-sampled to 400 Hz and zero-padded to generate signals of fixed length (12 × 4,096), used as input to the deep learning model.

### Deep learning modeling

We used a previously developed deep learning model to predict ECG-age (18). The model was based on 1,558,415 patients from the CODE study (26), which is part of the Telehealth Network of Minas Gerais, Brazil. The study collected ECGs in Brazilian primary care settings from 2010 to 2017 (27), and it represents one of the largest ECG databases in the world for AI-ECG applications (26). The ECG-age model uses a convolutional neural network with unidimensional signals. The network includes five residual blocks, each with two convolutional layers. More details could be found in the previous publication (18). The prediction model and the codes are available at GitHub (https://github.com/antonior92/ecg-age-prediction).

### Definitions of lifestyle factors

We considered four lifestyle factors, including cigarette smoking, alcohol consumption, physical activity, and diet, similar to a prior study (28). Smoking status was categorized into current smoker, or never/previous smokers. For physical activity, we used the American Heart Association recommendations of at least 150 min moderate intensity physical activity or 75 min of vigorous activity per week (or an equivalent combination) (29). Moderate alcohol consumption was defined as less than 14 grams per day women and less than 28 grams per day for men. Healthy diet was defined as the proper consumption of at least four out of seven commonly eaten food groups (30).

We assigned 1 point for a healthy level and 0 points for an unhealthy lifestyle. The sum of the healthy score ranged from 0 to 4, where a higher score indicates a higher adherence to healthy lifestyle components (28, 31). We also divided participants into three groups, with a score of 4 as an ideal lifestyle, scores 2 and 3 as an intermediate lifestyle, and 0 or 1 as an unfavorable lifestyle.

### Definitions of prevalent cardiovascular diseases and risk factors

The prevalent cases of atrial fibrillation, type 2 diabetes, heart failure, hypercholesterolemia, hypertension, myocardial infarction and stroke were defined by either self-report, ICD-9 or ICD-10 codes at the time or before the ECG examination. Body mass index (BMI) was calculated as weight in kilograms divided by height in meters squared, which were measured at the examination centers.

### Statistical analyses

Clinical variables were reported as mean ± standard deviation for continuous variables, and *n* (%) for dichotomous variables. The difference between ECG-age and chronological age was defined as Δage and corrected for chronological age (32). Participants were classified into three tertiles based on their Δage. Each tertile represents one aging group: first tertile for decelerated aging, second tertile for normal aging, and third tertile for accelerated aging. We used multivariable linear regression model to evaluate the associations of lifestyle factors and Δage adjusted for sex and age at the time when ECG was conducted. We also performed sex stratified analysis to assess the association of Δage with each lifestyle factor in men and women separately. In order to know potential mediating effects of ECG-age and sex, we added an additional analysis by including ECG-age*sex as the interaction terms. We further assessed the association of Δage with prevalent cardiovascular diseases and risk factors by logistic regression models adjusted for sex and age. In the sensitivity analysis, we defined extremely decelerated aging as those whose predicted ECG-age were more than 20 years younger than their chronological age, and extremely accelerated aging as those with predicted ECG-age were more than 20 years older than their chronological age. Statistical significance was claimed with two-sided *P* values less than 0.05. All the analyses were performed using R software package version 4.0.3 (https://www.r-project.org/).

## Results

**Figure 1:**
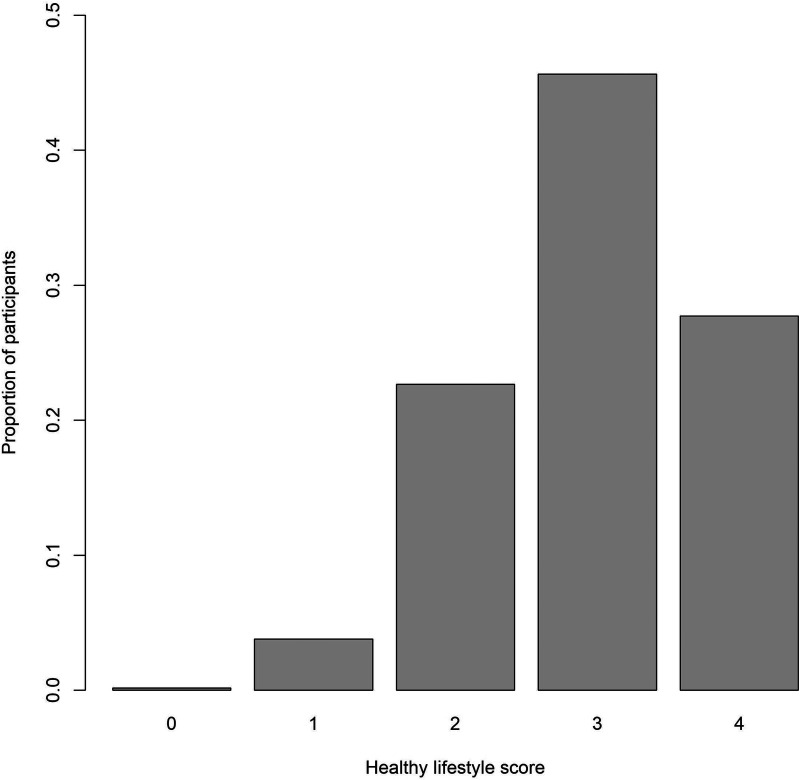
Association of predicted ECG-age with chronological age.

This study included 44,094 participants (mean age 64 ± 8, 51.4% women). The baseline clinical characteristics of participants are shown in Table 1. The detailed age distribution is depicted in Supplementary Figure S1. We observed a significant correlation between chronological age and ECG-age with a correlation coefficient of 0.54 (*P* < 0.001). The correlation between chronological age and ECG-age is shown in Figure 1. The median absolute error between ECG-age and chronological age after age adjustment was 9.1 ± 6.6 years (defined as Δage).

**Table 1:** Clinical characteristics of study participants (*N* = 44,094).

Characteristics*	Decelerated aging (*N* = 14,698)	Normal aging (*N* = 14,698)	Accelerated aging (*N* = 14,698)
Age, years	64 ± 8	65 ± 8	64 ± 8
Women, *n* (%)	8,633 (58.7)	7,472 (50.8)	6,451 (44.5)
SBP, mmHg	133 ± 18	137 ± 19	140 ± 19
DBP, mmHg	79 ± 10	82 ± 10	83 ± 11
BMI, kg/m2	25.8 ± 4.2	26.7 ± 4.4	26.8 ± 4.4
Atrial fibrillation, *n* (%)	170 (1.2)	259 (2.8)	437 (3.0)
Type 2 diabetes mellitus, *n* (%)	243 (1.7)	367 (2.5)	386 (3.3)
Heart failure, *n* (%)	38 (0.3)	50 (0.3)	93 (0.6)
Hypercholesterolemia, *n* (%)	1,872 (12.7)	2,361 (16.1)	2,568 (17.5)
Hypertension, *n* (%)	2,621 (17.8)	3,816 (26.0)	4,530 (30.7)
Myocardial infarction, *n* (%)	119 (0.8)	173 (1.2)	189 (1.3)
Stroke, *n* (%)	37 (0.3)	54 (0.4)	69 (0.5)
Healthy lifestyle scores	3.0 ± 0.8	3.0 ± 0.8	2.9 ± 0.8
Healthy diet, *n* (%)	7,029 (47.8)	6,677 (45.4)	6,492 (44.2)
No or moderate alcohol consumption, *n* (%)	10,086 (68.6)	9,987 (67.9)	9,625 (65.5)
Physically active, *n* (%)	12,949 (88.1)	12,804 (87.1)	12,648 (86.1)
Non-smoking, *n* (%)	14,298 (97.3)	14,249 (96.9)	14,083 (95.8)

Values are *n* (%) for dichotomous variables, mean ± standard deviation for continuous variables. Differences between tertiles were assessed by one-way ANOVA test for continuous variables or Pearson's chi-squared test for dichotomous variables. All of them were significant (*P *< 0.05).

*BMI, body mass index; SBP, systolic blood pressure; DBP, diastolic blood pressure.

**Figure 2:**
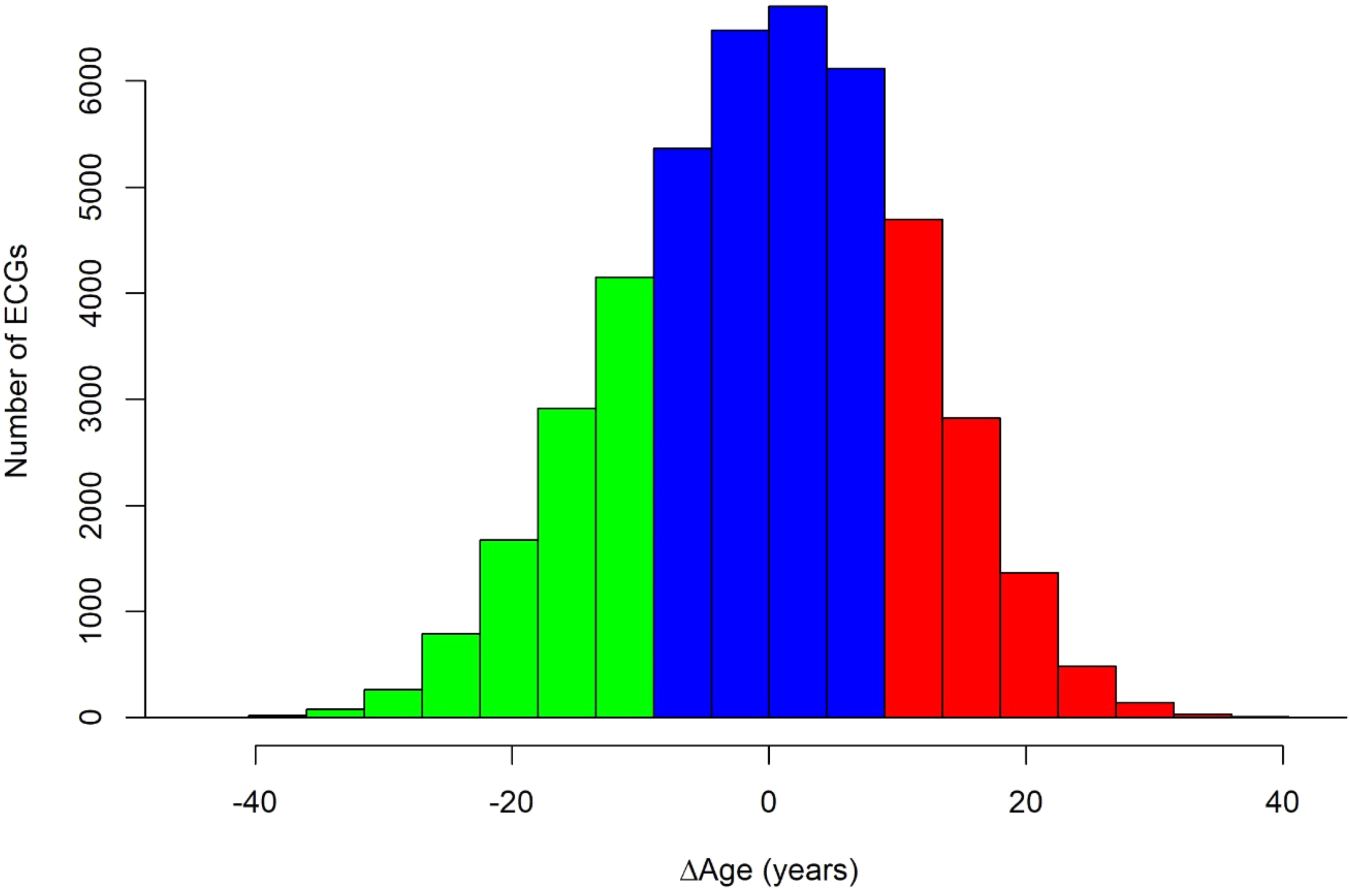
Distribution of the difference between chronological and ECG-age (Δage) among study participants. The figure is colored green for decelerated aging (first tertile), blue for normal aging (second tertile), and red for accelerated aging (third tertile).

We further classified participants into three tertiles based on their Δage (Figure 2), including accelerated aging, normal aging, and decelerated aging. As shown in Table 2, in comparison with normal aging, participants with accelerated aging are more likely to have atrial fibrillation [OR = 1.74, 95 confidence interval (CI) 1.48–2.03], type 2 diabetes (OR = 1.30, 95% CI 1.13–1.49), heart failure (OR = 1.84, 95% CI 1.30–2.60), hypercholesterolemia (OR = 1.11, 95% CI 1.04–1.18), and hypertension (OR = 1.28, 95% CI 1.22–1.35), but not myocardial infarction (*P *= 0.64) or stroke (*P *= 0.17). In contrast, participants with decelerated aging are less likely to have atrial fibrillation (OR = 0.76, 95% CI 0.63–0.93), type 2 diabetes (OR = 0.74, 95% CI 0.62–0.87), hypercholesterolemia (OR = 0.86, 95% CI 0.81–0.93), and hypertension (OR = 0.67, 95% CI 0.63–0.71).

**Table 2:** Association of accelerated aging and decelerated aging with prevalent cardiovascular disease and risk factors.

Clinical diagnosis	Accelerated aging			Decelerated aging		
	OR	95% CI	*P*	OR	95% CI	*P*
Atrial fibrillation	1.74	1.48–2.03	<0.001	0.76	0.63–0.93	0.01
Type 2 diabetes	1.30	1.13–1.49	<0.001	0.74	0.62–0.87	<0.001
Heart failure	1.84	1.30–2.60	<0.001	0.89	0.58–1.36	0.58
Hypercholesterolemia	1.11	1.04–1.18	0.001	0.86	0.81–0.93	<0.001
Hypertension	1.28	1.22–1.35	<0.001	0.67	0.63–0.71	<0.001
Myocardial infarction	1.05	0.85–1.29	0.64	0.84	0.66–1.06	0.14
Stroke	1.29	0.90–1.84	0.17	0.79	0.52–1.21	0.28

Decelerated aging: Participants with the first tertile Δage; normal aging: participants with the second tertile Δage; accelerated aging: participants with the third tertile of Δage (ECG-age minus chronological age).

OR, odds ratio comparing to normal aging; CI, confidence interval.

In the sensitivity analysis, we examined participants whose predicted ECG-age were more than 20 years different from their chronological age. They were defined as extremely decelerated aging (Δage < 20 years, *n* = 1,952) and extremely accelerated aging (Δage > 20 years, *n* = 1,261). As shown in Supplementary Table S1, participants with extremely accelerated aging are more likely to have atrial fibrillation, type 2 diabetes, heart failure, hypercholesterolemia, and hypertension. In contrast, participants with extremely decelerated aging are less likely to have atrial fibrillation, hypercholesterolemia, hypertension, and myocardial infarction.

### Association of ECG-age with lifestyle factors

**Figure 3:**
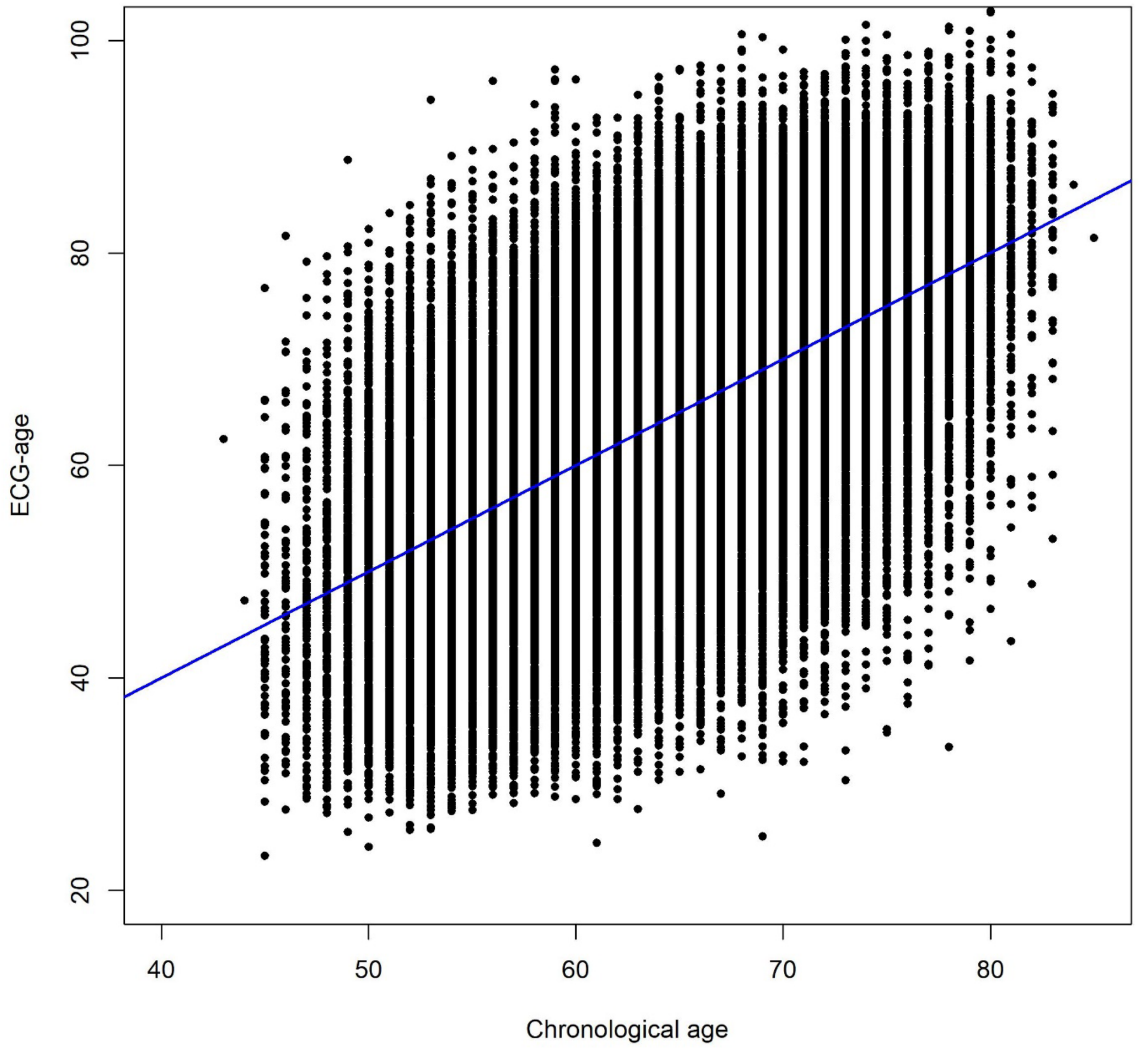
Distribution of healthy lifestyle scores among participants. Each point represents the adherence of one healthy lifestyle factor.

Table 1 shows the proportion of participants engaged in each healthy lifestyle factor, ranging from non-smoking (96.7%) to a healthy diet (45.8%). Nearly three quarters of participants (73.4%) engaged in 3 of 4 healthy lifestyle factors (Figure 3). Among all participants, 27.7% had an ideal lifestyle (scores 4), whereas 4.0% had an unfavorable lifestyle (scores 0 and 1).

We then tested the association of each lifestyle factor with Δage. As shown in Table 3, all four healthy lifestyle factors were significantly associated with decreased ECG-age. The most significant association was observed for non-smoking, which was associated with 2.37 years [standard error (SE) 0.30] younger ECG-age. Each point of overall lifestyle score was also associated with 0.75 years (SE 0.07) ECG-age.

**Table 3:** Association of Δage with overall lifestyle score and each individual lifestyle factor.

Lifestyle factor	β	SE	*P*
Healthy Diet	−0.41	0.11	<0.001
No or moderate alcohol consumption	−0.95	0.11	<0.001
Physically active	−1.04	0.16	<0.001
Non-smoking	−2.37	0.30	<0.001
**Overall lifestyle score**	−0.75	0.07	<0.001

β, years of decrease of predicted ECG-age; SE, standard error.

We also performed sex stratified analysis. As shown in Supplementary Table S2, Δage was significantly associated with all lifestyle factors for both men and women. For all lifestyle factors, the effect size was larger in men compared with women, and the difference was significant for alcohol consumption (interaction *P *= 0.004) and smoking (interaction *P *= 0.04).

We further examined the association of Δage with different lifestyle categories. People with an ideal lifestyle tended to be 2.50 years (SE = 0.29) younger than those with an unfavorable lifestyle. In addition, participants with unfavorable lifestyles are more likely to have accelerated aging compared to those with a favorable lifestyle (OR = 1.14, 95% CI 1.08–1.19).

## Discussion

In this large middle-aged to old population in the UK, we predicted biological age from the ECG and assessed its association with lifestyle factors. The predicted ECG-age is significantly correlated with chronological age. We also found that the difference between ECG-age and chronological age was associated with all of four studied lifestyle factors as well as prevalent cardiovascular diseases and risk factors.

Biological age can be affected by genetic factors and many comorbid conditions, such as heart failure, hypertension and coronary artery disease (17). As healthy lifestyles are associated with lower mortality and CVD risk across different socioeconomic subgroups (31), it would be important to know the effects of lifestyle factors on biological age. A wide range of mechanisms might be involved modifying the aging process, such as increased cerebral blood flow and reduced oxidative damage (33–36). Some lifestyle factors are also interrelated (37, 38), so it is possible to combine multiple lifestyle factors to investigate their integrative effects on aging. This study underscores the importance of healthy lifestyle on biological aging, which might facilitate future implementation of preventive interventions to promote healthy behaviors.

Our study supports broadly the notion that deep learning models using 12-lead ECG provide important predictive usefulness for biological age. The model was trained in the Brazil cohort but still showed strong correlation with chronological age in the current cohort with most European participants. The deep learning algorithms are purely data-driven without manually extracted features. On the other hand, the exact morphological characteristics contributing to the prediction remain to be investigated. Some studies using saliency mapping and median waveform analysis would help identify regions of ECGs critically contributing to the prediction (15). Future advances in interpretable machine learning might enable a better illustration of the decision process.

It is worth to note that the deep learning model used in the current study was developed based on ECG records from 1,558,415 participants in the state of Minas Gerais (Brazil) (18). On the other hand, the majority of UK Biobank participants are of European ancestry. Interestingly, a similar Δage was observed between two studies despite differences in age ranges and ancestries. Our results suggest that prevalent cardiovascular diseases and risk factors might play a more important role in ECG-age compared with ancestries. Therefore it would be important to share ECGs across different health conditions even with different ancestries, which would enable future development of more sophisticated deep learning models to predict biological age. A broader and more detailed plan for data sharing was also underscored in the recently released policy from the National Institutes of Health (https://grants.nih.gov/grants/guide/notice-files/NOT-OD-21-013.html).

With the aging population and increasing burden of age-related diseases, it would be useful to implement a convenient and low-cost approach to assess aging in clinical settings. Given the wide availability of ECG, deep learning predicted ECG-age could provide a promising solution for the estimation of biological age. Such a tool might be incorporated into standard ECG management platforms as an additional feature to reflect an individual's overall health. New wearable devices are also being developed to perform ECG even easier and more cost-effective (39, 40). A future extension of our work is to assess aging using single-lead ECG collected from wearable devices, which could be used to further investigate the effects of short and long-term lifestyle changes on ECG-age in real time. All these efforts would help guide better preventive efforts and clinical screening of people with high risk of age-related diseases.

We acknowledge several limitations of our study. The majority of participants in UK Biobank are of European ancestry, therefore our findings may not be generalizable to other race/ethnic groups. There is also potential selection bias as many participants are relatively healthy with high socioeconomic status. In addition, it is unclear if there is any different pattern for people at a younger age or older age outside the range of our study population. We included four self-reported lifestyle factors in the current study, which might not capture all relevant lifestyle information. For example, sleep is probably an important factor. These lifestyle factors were also not cross-validated by medical records. We did not perform an exhaustive search of comorbidities so there could be unmeasured confounding factors that were not taken into account. Moreover, we observed participants with prevalent cardiovascular diseases or risk factors tended to have accelerated aging. However, this does not necessarily mean that accelerated aging would increase their future risk. Future studies combining classical machine learning and deep learning models might reach a better performance (11). Additional important questions that cannot be answered by our analysis is the stability of the ECG-age over the short-term, change over time (long-term), and reversibility.

In conclusion, we estimated biological age by ECG and assessed its association with lifestyle factors in a large population cohort. A strong correlation was observed between the presence of all four healthy lifestyle factors and deaccelerated aging, which underscores the importance of a healthy lifestyle to reduce the burden of aging-related diseases. Future work is needed to better understand the molecular mechanism underlying lifestyle on healthy aging.

## Data Availability

The original contributions presented in the study are included in the article/**Supplementary Material**, further inquiries can be directed to the corresponding author/s.
