# Supplementary material for: Association of lifestyle with deep learning predicted electrocardiographic age
Source: Front Cardiovasc Med. 2023 Apr 24;10:1160091. doi: 10.3389/fcvm.2023.1160091 (PMC10165078; doi:10.3389/fcvm.2023.1160091)
Supplement: Supplementary file 1 [file Datasheet1.pdf]

Supplemental Figure 1. Distribution of chronological age among study participants (median 65 years old).

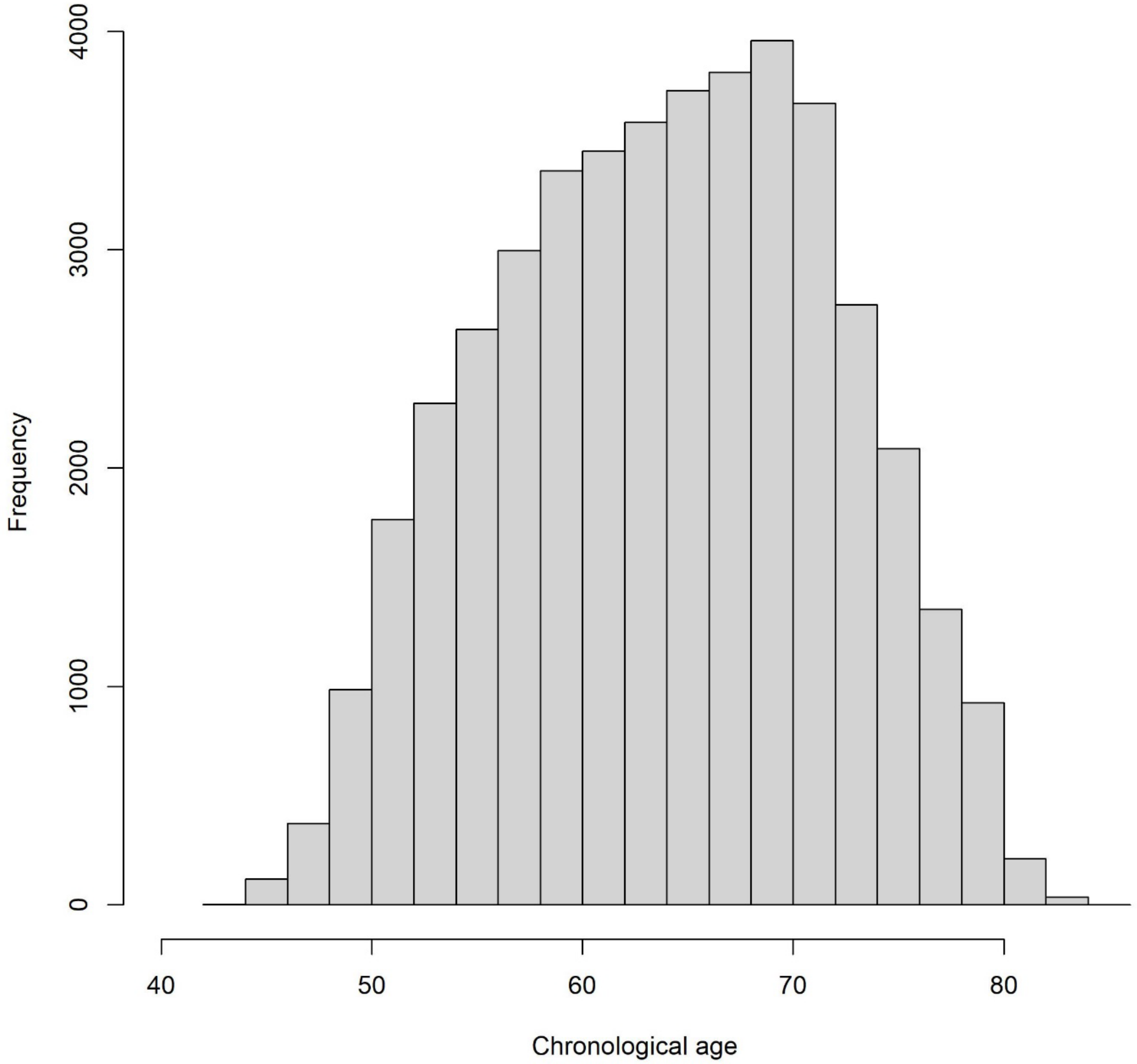

Supplemental Table 1. Association of extremely accelerated aging and extremely decelerated aging with prevalent cardiovascular disease and risk factors

Disease	Extremely accelerated aging			Extremely decelerated aging		
	**OR**	**95% CI**	***P***	**OR**	**95% CI**	***P***
Atrial fibrillation	2.63	1.94-3.54	<0.001	0.60	0.39-0.92	0.02
Type 2 diabetes	1.58	1.15-2.16	<0.001	0.79	0.57-1.11	0.17
Heart failure	4.28	2.56-7.14	<0.001	0.68	0.28-1.67	0.40
Hypercholesterolemia	1.12	0.95-1.32	0.001	0.72	0.63-0.84	<0.001
Hypertension	1.58	1.39-1.79	<0.001	0.50	0.44-0.57	<0.001
Myocardial infarction	1.16	0.68-1.99	0.58	0.35	0.17-0.74	0.01
Stroke	1.84	0.85-3.96	0.12	0.43	0.14-1.34	0.15

OR: odds ratio compared to normal aging; CI: confidence interval
Extremely decelerated aging: Participants whose predicted ECG-age was more than 20 years younger than their chronological age; Extremely accelerated aging: Participants whose predicted ECG-age was more than 20 years older than their chronological age.

Supplemental Table 2. Sex-stratified analysis of the association of Δage with lifestyle factors.

Lifestyle factor	Men			Women			Sex interaction
	β	SE	*P*	β	SE	*P*	*P*
Healthy diet	-0.51	0.16	<0.001	-0.33	0.15	0.03	0.24
No/moderate alcohol consumption	-1.28	0.17	<0.001	-0.66	0.16	<0.001	0.004
Physically active	-1.18	0.23	<0.001	-0.96	0.22	<0.001	0.48
Non-smoking	-2.81	0.39	<0.001	-1.77	0.46	<0.001	0.04
Overall lifestyle score	-0.94	0.09	<0.001	-0.59	0.09	<0.001	0.002

ECG: electrocardiographic; β: years of decrease of predicted ECG-age; SE: Standard error
